# Supplementary material for: Evaluation of Agronomic Characteristics, Disease Incidence, Yield Performance, and Aflatoxin Accumulation among Six Peanut Varieties (Arachis hypogea L.) Grown in Kenya
Source: Toxins (Basel). 2023 Jan 28;15(2):111. doi: 10.3390/toxins15020111 (PMC9962968; doi:10.3390/toxins15020111)
Supplement: Supplementary file 1 [file toxins-15-00111-s001.zip › toxins-2116376-supplementary.pdf]

Method Validation

Various method validation parameters were evaluated they are standards linearity, recovery, accuracy, precision Limit of quantification (LOD), Limit of quantification (LOQ)

Linearity of standards was evaluated by consecutively injecting standards ranging from 0.52-104.1 ng/ml for each of the toxins at 6 levels. Spike recovery was performed at 3 levels (0.5MRL, 1MRL, and 2MRL) for each of the toxin i.e. 7.5,15 and 30 ng/g in six replicates. See data in table 1 below

Table S1: Summary of linearity and results from spike recovery (n=6)

Toxin	Calibration standards Linearity	Spike recovery				
	Range	Level 1 7.5ng/g	Level 2 15ng/g	Level 3 30ng/g	Average spike recovery	Spike recovery standard deviation
AFB1	0.52-104.1	77.6	74.01	74.43	75.36	1.63
AFB2	0.52-103.9	74.06	75.65	74.14	74.62	0.73
AFG1	0.52-103.9	102.32	84.18	76.52	87.67	10.82
AFG2	0.52-104.1	58.16	75.20	80.92	71.42	9.67
Total aflatoxins						

Evaluation of the method accuracy was performed by running QC (T04422QC peanut slurry) material obtained from Fera Science Ltd (Sand Hutton, York. UK) and past proficiency test sample from AOCS i.e. sample 5 and sample 6 of 34 aflatoxin peanut paste test 20220-2021. The data is shown in table 2 and 3 below.

Table S2: comparison between assigned value and measured value of quality control material T04422QC peanut slurry obtained from Fera science

**Aflatoxin**	**Assigned value (ng/g)**	**Range (ng/g)**	**Measured value (n-6) (ng/g)**
Aflatoxin B1	3.75	2.10-5.40	3.44±0.65
Aflatoxin B2	2.75	1.54-3.96	2.82±0.46
Aflatoxin G1	1.85	1.04-2.67	1.51±0.21
Aflatoxin G2	1.73	0.97-2.49	2.21±0.32
Total aflatoxin	10.0	5.6-14.4	9.97±0.84

Table S3: comparison between assigned value and measured value of AOCS sample 5 and sample 6 of 34 aflatoxin peanut paste test 20220-2021

**Sample**	**Toxin**	**Consensus value (ng/g)**	**Range (ng/g)**	**Measured value (ng/g)**
Sample 5	Total aflatoxin	14.85	7.96-21.73	21.35
Sample 6	Total aflatoxin	10.2	5.4-15.0	14.21

Additionally, the method was subjected to proficiency testing for the year 2022 and satisfactory results were obtained as illustrated in table 3 below.

Table S4: Summary of proficiency testing results; Aflatoxins in peanut (water/Nut slurry)

Analyte/Toxin	Laboratory Reported value µg/kg	PT assigned value µg/kg	Lab z-Score	PT Range value µg/kg	FAPAS PT04453
Aflatoxin B1	4.24	4.72	-0.5	2.62-6.79	
Aflatoxin B2	3.18	3.49	-0.4	1.95-5.02	
Aflatoxin G1	1.68	2.38	-1.3	1.34-3.43	
Aflatoxin G2	1.89	1.68	0.6	0.943-2.42	
Total Aflatoxin	10.99	12.3	-0.5	6.87-17.7	

Limit of detection and limit of quantification were determined from the data obtained from the six replicate measurements of the quality control material **T04422QC peanut slurry** using the standard deviation method as recommended by B. Magnusson and U. Ornemark. (2014)

Data on the method LOD and LOQ are shown in table 5

Table S5: LOD and LOQ method specifics

Toxin	LOD ng/g	LOQ ng/g
Aflatoxin B1	0.79	2.639
Aflatoxin B2	0.56	1.86
Aflatoxin G1	0.26	0.88
Aflatoxin G2	0.40	1.32
Total aflatoxin	1.03	3.42

Table S6: Percentage of positive samples analyzed for aflatoxins

Variety	Aflatoxin B1	Aflatoxin B2	Aflatoxin G1	Aflatoxin G2	Total Aflatoxins
CG7	50	0	50	100	100
CG9	0	0	50	50	75
CG12	50	25	75	75	75
ICGV-SM 90704	25	0	50	100	100
12991	50	50	100	100	100
Homabay Local (Control)	0	100	100	100	100

Table S7: Calibration information

Toxin	Retention time (min)	Calibration range (ng/ml)	Calibration equation	Coefficient of correlation
AFG2	4.125	104.1-0.521	Y = (12116.2)X + (0)	0.9995
AFG1	4.991	103.9-0.520	Y = (1734.77)X + (0)	0.9996
AFB2	6.225	103.9-0.520	Y = (13358.7)X + (0)	0.9996
AFB1	7.686	104.10.5205	Y = (2929.36)X + (0)	0.9995

Figure S1: AFG2 calibration curve

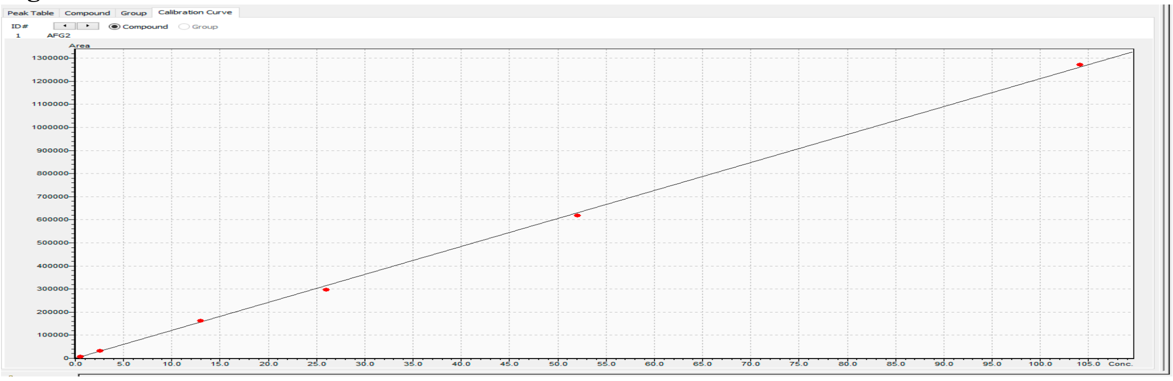

Figure S2: AFG1 calibration curve

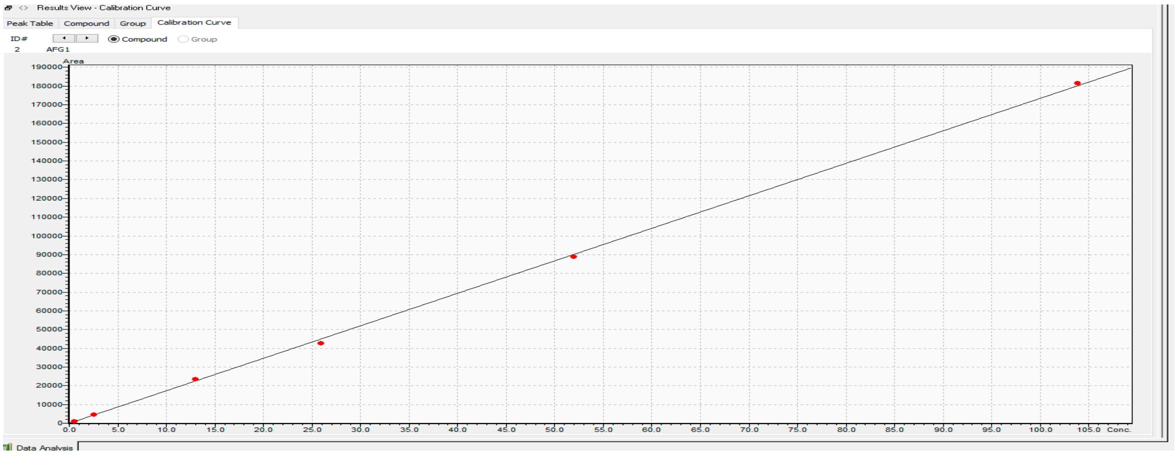

Figure S3: **AFB2 calibration curve**

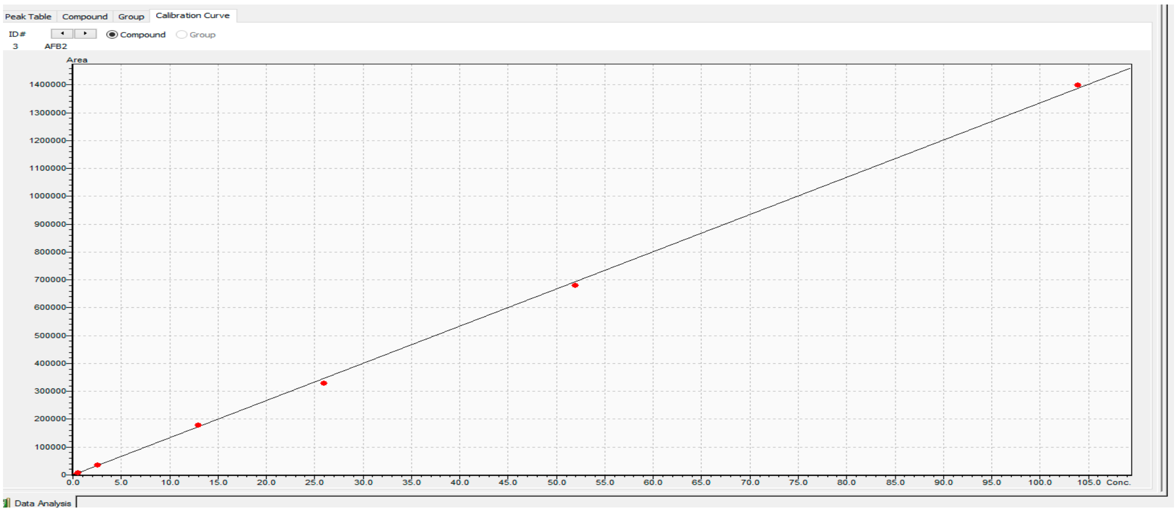

Figure S4: AFB1 calibration curve

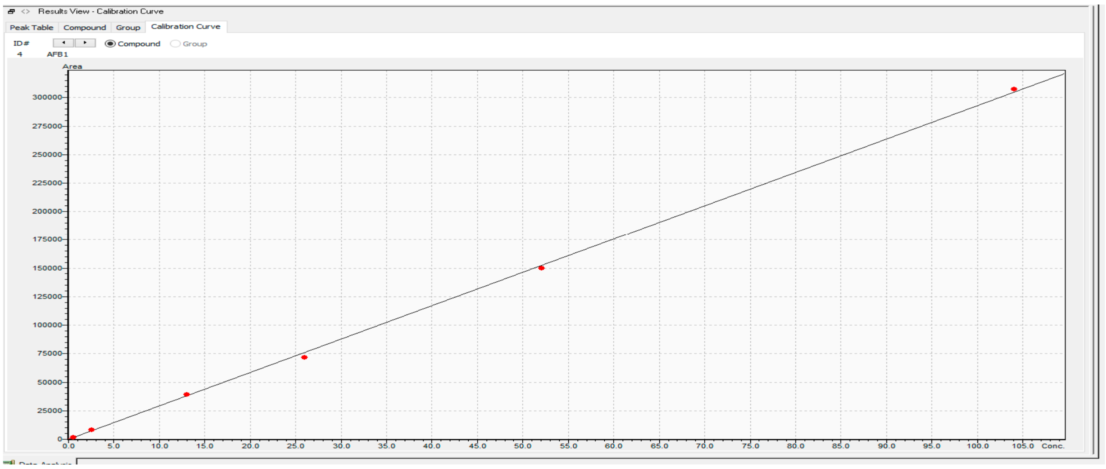

Figure S5: Over-layed chromatogram of simple and standard

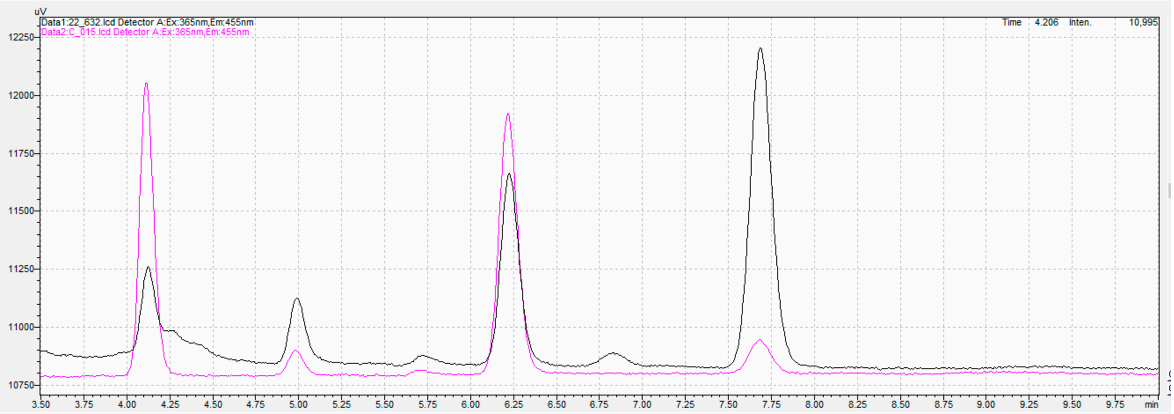

Key: Red chromatogram – calibration standard

Black chromatogram- sample extract

Table S8: Abbreviation List

Abbreviation	Definition
ACR	Aspergillus crown rot
AEZ	Agro ecological zones
AFB1	Aflatoxin B1
AFB2	Aflatoxin B2
AFG1	Aflatoxin G1
AFG2	Aflatoxin G2
ANOVA	Analysis of Variance
Ca	Calcium
ELS	Early leaf spot
Fe	Iron
GRV	Groundnut Rosette Virus
HSD	Honestly significant difference
ICRISAT	Crops Research Institute for the Semi-Arid Tropics
LLS	Late leaf spot
M.A.S.L.	Meters above sea level
Mg	Magnesium
PDA	Potato Dextrose Agar
PPB	Parts per billion
PTFE	**Polytetrafluoroethylene**
UHPLC-FLD	Ultra High Performance Liquid Chromatography and Fluorescence Detection
Zn	Zinc

Table S9: List of Materials and Reagents

Newly improved peanut varieties (CG7, CG12, CG7, ICGV-SM 90704	ICRISAT Malawi
Locally used varieties (12991, Homabay Local)	Homabay
Animal manure	Locally sourced (Nyakach)
Ridomil Gold	Syngenta
Duduthrin 1.75EC	Twiga chemicals
Ethanol	Scharlau
Methanol	Scharlau
Sodium hypochlorite	Insulation world Kenya
Distilled water	Scharlau
Potato Dextrose Agar (PDA)	Hi media
Weighing balance	Sartorius
Super pro moisture analyzer	Supertech agroline
Grinder	Herbs and spices
Drying canvas	Locally made
Airtight plastic containers	Afro plastics limited
Polypropylene centrifuge tube	BD, Franklin Lakes, NJ, USA
Falcon tubes	BD, Franklin Lakes, NJ, USA
Mechanical orbital shaker	New Brunswick, NJ, USA
PTFE syringe filter	New Brunswick, NJ, USA
Nexera UHPLC system	(Shimadzu Corporation, Kyoto, Japan)
A Synergi Hydro-RP analytical column	(Phenomenex, Torrance, CA, USA)
LabSolutions software version 5.89	(Shimadzu Corporation, Kyoto, Japan, 2014)
